# Efficacy of structured teaching program for rehabilitation of children with autism spectrum disorder: A systematic review and meta-analysis

**DOI:** 10.12669/pjms.41.9.12634

**Published:** 2025-09

**Authors:** Yinfang Zhu, Manyan Zhang, Dandan Ma, Pingping Wang

**Affiliations:** 1 Yinfang Zhu Department of Children’s Rehabilitation, the Affiliated Hospital of Shaoxing University, Shaoxing, Zhejiang Province 312000, P.R. China; 2 Manyan Zhang Department of Children’s Rehabilitation, the Affiliated Hospital of Shaoxing University, Shaoxing, Zhejiang Province 312000, P.R. China; 3 Dandan Ma Department of Children’s Rehabilitation, the Affiliated Hospital of Shaoxing University, Shaoxing, Zhejiang Province 312000, P.R. China; 4 Pingping Wang Department of Neurological Rehabilitation, the Affiliated Hospital of Shaoxing University, Shaoxing, Zhejiang Province 312000, P.R. China

**Keywords:** Autism, Meta-Analysis, Psychology, Rehabilitation, TEACCH

## Abstract

**Background & Objective:**

The Structured Teaching Program, based on the Treatment and Education of Autistic and Related Communication Handicapped Children (TEACCH) program approach, has been a cornerstone in the educational rehabilitation of children with autism spectrum disorder (ASD). This study assesses the efficacy of structured teaching program in managing ASD children.

**Methodology:**

Systematic search of Medline, Google Scholar, ScienceDirect, Cochrane Central Register of Controlled Trials and trial registries was done for trials comparing structured teaching program with no treatment, alternative treatments, or as part of multimodal intervention in children with ASD from inception of database till end date of search (31 January 2025). We used Cochrane risk of bias-2 tool and risk of bias tool for non-randomized trials to assess quality of studies and effect sizes were calculated using standardized mean differences (SMD) with 95% confidence interval (CI).

**Results:**

Pooled analysis included 10 studies. Significant (p<0.05) improvements were observed in fine (SMD=0.45; 95% CI: 0.12 to 0.79) and gross motor skills (SMD=0.54; 95% CI: 0.21 to 0.88). High heterogeneity was found across most outcomes. However, no significant improvements were noted in communication (SMD=-0.33; 95% CI: - 1.99 to 1.33), socialization (SMD=0.12; 95% CI:- 0.61 to 0.85), daily living (SMD=-0.09; 95% CI:- 1.62 to 1.44) and cognitive performance (SMD=0.07; 95% CI:- 0.43 to 0.57).

**Conclusion:**

The structured teaching program shows potential in improving motor skills in children with ASD but does not consistently enhance other developmental domains. Personalizing structured teaching program to individual needs and integrating it with other therapies may be necessary to maximize its efficacy.

**Study registration:**

PROSPERO, CRD42023480468

## INTRODUCTION

Autism Spectrum Disorder (ASD) refers to individuals with impaired social communication, repetitive behaviours and restricted interests.^1^ The condition manifests in early childhood and impacts on many aspects of patient’s functioning.^1^ There is a constant pressing need for effective evidence-based educational interventions that cater specifically to the learning styles and needs of children with ASD.^2^

The Treatment and Education of Autistic and Related Communication Handicapped Children (TEACCH) program, developed in the 1970s, is a pioneering structured teaching approach for rehabilitation of children with ASD^3^ that was designed to specifically address the unique challenges associated with the condition. TEACCH is based on the understanding that individuals with autism have distinct learning profiles and cognitive styles.^4^ This perspective has given rise to educational practices that aim to capitalize on the strengths of children with autism, while simultaneously supporting their areas of difficulty.

Structured teaching, the cornerstone of the TEACCH program, encompasses a number of strategies including physical organization, individualized schedules, work systems and visual structure.^5^ The program is tailored to promote engagement, reduce distractions and anxiety, improve task organization and transition and extensively uses visual supports to convey information, sequence tasks and clarify expectations, tapping into the typically strong visual processing skills of individuals with autism. The philosophy of TEACCH extends beyond academic achievements and emphasizes fostering independence and improving life skills, which are critical components of a holistic approach to autism intervention.^5^

Numerous studies have demonstrated the efficacy of TEACCH program in enhancing the educational outcomes for children with autism.^6^-^9^ While the program is not designed as a standalone therapy for ASD, it is often integrated with other treatment modalities, thus contributing to a comprehensive intervention strategy.

Despite accumulating evidence on structured teaching programs such as TEACCH, the literature remains marked by several unresolved issues. Controlled trials have reported gains in adaptive behaviours and cognitive skills, yet quasi-experimental studies often show inconsistent effects on communication and social functioning. Moreover, few investigations have systematically evaluated how factors such as intervention duration, delivery setting (e.g., school versus clinic), or age at initiation influence outcomes. This variability in study design, outcome measures, and participant characteristics has left clinicians and educators uncertain about optimal program parameters. By synthesizing data across study types and stratifying results by key intervention features, our review aimed to clarify these contradictions and identify the conditions under which structured teaching is most effective.

To conduct systematic review and meta-analysis of structured teaching programs for children with autism spectrum disorder, evaluating pooled effect sizes on core developmental domains and exploring the impact of intervention characteristics on treatment efficacy.

## METHODOLOGY

A thorough search of Medline, Google Scholar, ScienceDirect and the Cochrane Central Register of Controlled Trials (CENTRAL) databases and clinical trial registries (ClinicalTrials.gov and the WHO International Clinical Trials Registry Platform) was done without any language restrictions from the establishment of each database until January 2025. The end date of search was 31 January 2025. The terms used in our search were “structured teaching program”, “TEACCH”, “Autism”, “Autism spectrum disorder”, “randomized controlled trial”, “clinical trial”, “controlled clinical trial” in various permutations and combinations. Detailed search strategy is provided in the supplementary appendix**.** Bibliographies of retrieved trials were manually searched for additional studies. Authors of the research papers were contacted to obtain any missing information required for our evaluations.

### Inclusion criteria:

Randomized controlled trials (RCTs), quasi-experimental trials and other forms of trials with control group; Studies conducted in children with autism spectrum disorder and comparing the effectiveness of structured teaching program in the form of TEACCH program against standard or usual care.

### Exclusion criteria:

Studies with the pre-post design, observational studies; Studies with no comparator group.

The outcomes assessed were as follows: communication, socialisation, daily living, motor skills, adaptive behaviour, imitation, fine motor and gross motor skills, perception, cognitive performance, eye-hand coordination, cognitive verbal performance and parental stress index.

### Data collection & analysis:

Titles, abstracts and keywords of articles were independently examined by two researchers (YZ and MZ) and full-texts of potentially relevant articles were independently evaluated by the lead and secondary researchers (YZ and MZ) based on our predetermined criteria. Any differences were resolved by discussion or by consulting a third researcher (DM). Our reporting of the review adhered to the PRISMA guidelines.^10^

### Data extraction:

The lead researcher (YZ) compiled key variables from the studies, such as the date of data extraction, the title of the trial and its authors. The recorded methodological parameters included the type of study, participant demographics and the research context; detailed participant information including the number of the samples and criteria for inclusion or exclusion; details of the intervention; lengths of follow-ups; and primary and secondary results from each group at various assessment points.

### Risk of bias:

For randomized controlled trials (RCTs), the Cochrane risk-of-bias tool for randomized trials (RoB 2)^11^ was used to assess the likelihood of bias arising from the randomization, variability in interventions, missing data, outcome measurement and selection of the reported result.

For non-randomized studies of interventions (NRSI), we used the Risk of Bias in Non-randomized Studies - of Interventions (ROBINS-I) tool^12^ to systematically assess the risk of bias due to confounding, participant selection, classification of interventions, deviations from intended interventions, missing data, measurement of outcomes and reported results.

Risk of bias was classified as ‘low,’ ‘high,’ or ‘some concerns’. Each study was independently reviewed by two investigators (DM and PW). All differences were resolved by discussion or by talking to a third researcher (MZ).

### Statistical analysis:

STATA software was used to synthesize data from the included studies. For all the continuous outcomes, we recorded the means and standard deviations at the point of follow-up or study conclusion and calculated pooled estimates of these figures. These pooled estimates were then expressed as standardized mean differences (SMDs) along with their 95% confidence intervals (CI). Random effects model was employed for all the outcomes.^13^ Heterogeneity between the studies was assessed by Chi-square test and I^2^ statistic. Value more than 75% in I^2^ statistic was indicative of substantial heterogeneity. Forest plots were generated to visually present both individual study estimates and combined effect estimates.

To evaluate the reporting bias, we examined whether each trial had been registered and whether the complete trial protocol was accessible. We compared the outcomes listed in the protocol with those reported in the publication. Due to the inclusion of fewer than 10 trials in our review, we were unable to conduct a comprehensive assessment of publication bias.

## RESULTS

### PRISMA flowchart:

**Fig.1:**
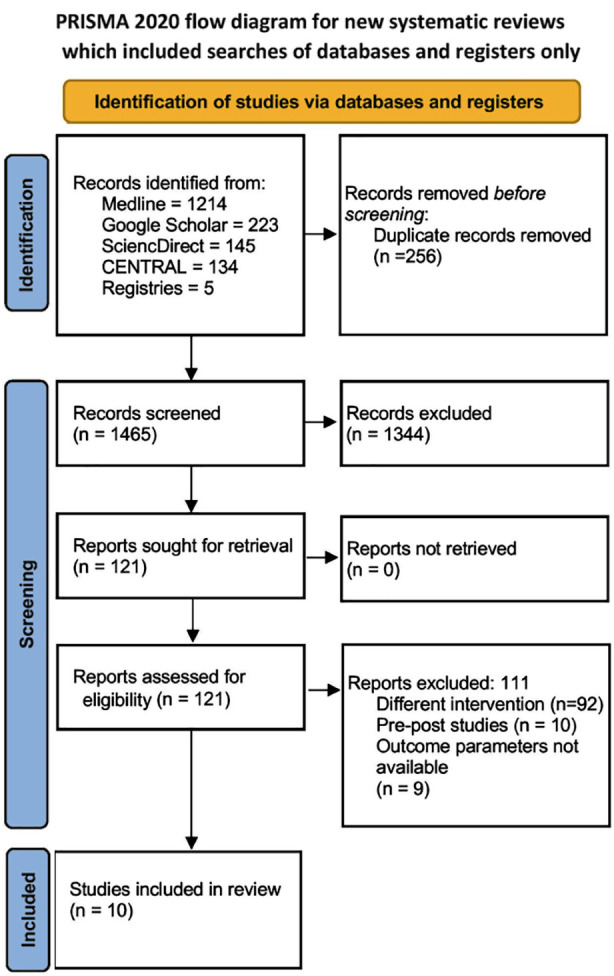
PRISMA flow diagram.

The detailed PRISMA flowchart of the study and the screening process is summarized in Fig.1. Overall, 1721 studies were obtained during the initial search across all databases. Of them, 121 full texts underwent screening for eligibility and 10 studies were included in the analysis.^6^-^9^,^14^-^19^

### Characteristics of the included studies:

Table-I shows the characteristics of the included studies. There was equal distribution of the RCTs and non-randomized trials across several countries, including Japan, Ireland, the USA, Italy and China. Participants were predominantly male individuals (76.4% to 100%) in all studies, reflecting the higher autism diagnosis rates in males. Sample sizes varied considerably, with the smallest trial having 11 participants and the largest 60. The interventions also differed in terms of intensity and duration, from as little as one hour per week over four weeks to up to 35 hours per week for twelve weeks. The mean age of participants ranged from 2.5 to 9.1 years, indicating that most studies focused on early childhood. Risk of bias assessment showed that three studies had lower risk, three studies had some concerns and four studies had higher risk of bias (Supplementary Table-I & II).

**Table-I:** Details of included studies.

Study	Nature	Location	Participant diagnosis	Sample size	Intervention intensity and duration	Assessment tool	Mean age (in years)	Male (% of total participants)	Risk of bias
Ichikawa (2013)	RCT	Japan	High functioning autism	TEACCH=5 Control group=6	2 hours per week for 6 months	Parental stress index	5.3	80	Some concerns
McConkey (2010)	Non-randomized trial	Ireland	Autism	TEACCH=35 Control group=26	1.5 hours per week for 10 weeks	PEP-R, VABS, GARS	2.8	90	Low risk
Nowell (2019)	RCT	USA	Autism	TEACCH=8 Control group=9	NR	COP	6.75	76.4	Some concerns
Ozonoff (1998)	Non-randomized trial	USA	Autism	TEACCH=11 Control group=11	1 hour per week for 4 weeks	PEP-R	4.4	82	High risk
Paneroi (2002)	Non-randomized trial	Italy	Autism with comorbid intellectual disability	TEACCH=13 Control group=10	30 hours per week for 12 weeks	PEP-R, VABS	9.1	100	High risk
Paneroi (2009)	Non-randomized trial	Italy	Autism with comorbid intellectual disability	TEACCH=11 Control group=10	36 weeks	PEP-R, VABS	8.7	100	High risk
Tsang (2007)	Non-randomized trial	China	Autism with pervasive development disability	TEACCH=18 Control group=16	35 hours per week for 12 weeks	C-PEP; HK-VABS; M-P-R	4.6	94	Low risk
Turner-Brown (2019)	RCT	USA	Autism	TEACCH= Control group=	90 minutes in home session for 6 months	Parental stress index	2.8	85.7	Low risk
Welterlin (2012)	RCT	USA	Autism	TEACCH=10 Control group=10	14 weeks	MSEL; SIB-R	2.5	90	High risk
Zeng (2021)	RCT	China	Autism	TEACCH=30 Control group=30	2.5 hours per week for 24 weeks	C-PEP	4.8	78.3	Some concerns

***Abbreviation:*** NR - Not reported; USA - United States of America; RCT - Randomized controlled trial; C-PEP = Chinese Psychoeducational Profile;

VABS = Vineland Adaptive Behavior Scales; GARS = Gilliam Autism Rating Scale; HK-VABS = Hong Kong Based VABS;

M-P-R = Merrill-Palmer—Revised; MSEL = Mullen Scales of Early Learning; PEP-R = Psychoeducational Profile Revised;

SIB-R = Scales of Independent Behavior—Revised; COP - Child Observation Protocol.

**Supplementary Table-I:** Risk of bias assessment for RCTs.

Study	Randomization	Deviation from intended intervention	Missing outcome data	Measurement of outcome	Selective reporting of result	Risk of bias
Ichikawa (2013)	Low	Some concerns	Low	Low	Some concerns	Some concerns
Nowell (2019)	Low	Some concerns	Some concerns	Low	Low	Some concerns
Turner-Brown (2019)	Low	Low	Low	Low	Low	Low risk
Welterlin (2012)	Low	High	High	Low	Some concerns	High risk
Zeng (2021)	Low	Low	Some concerns	Low	Some concerns	Some concerns

**Supplementary Table-II:** Risk of bias assessment for non-RCTs.

Study	Confoudning	Selection	Classification of intervention	Deviation from intended intervention	Missing outcome data	Measurement of outcome	Selective reporting of result	Risk of bias
McConkey (2010)	Low	Low	Low	Low	Low	Low	Low	Low risk
Ozonoff (1998)	High	Some concerns	Low	Some concerns	Low	Low	Some concerns	High risk
Paneroi (2002)	Some concerns	Low	Some concerns	Low	High	Some concerns	Some concerns	High risk
Paneroi (2009)	High	Low	Some concerns	Some concerns	High	Some concerns	Some concerns	High risk
Tsang (2007)	Low	Low	Low	Low	Low	Low	Low	Low risk

### Communication:

**Fig.2:**
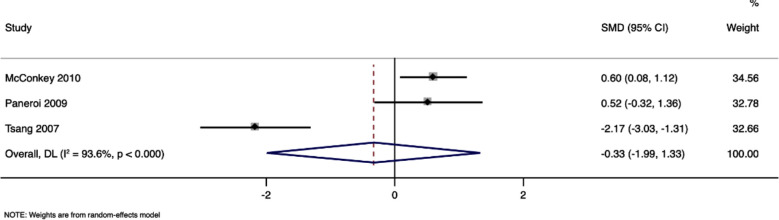
Forest plot showing the difference in communications scores between intervention and control arms.

Three studies with 118 participants have reported the communication scores in children who received TEACCH program and children who got standard care, with a pooled SMD of -0.33 (95%CI, -1.99 to 1.33) and comparable in both groups (p=0.70). Fig.2 The I^2^ value was 93.6% indicating a high level of heterogeneity. Sensitivity analysis did not reveal any significant effect on the pooled estimate, indicating robustness of the results.

### Socialization:

**Fig.3:**
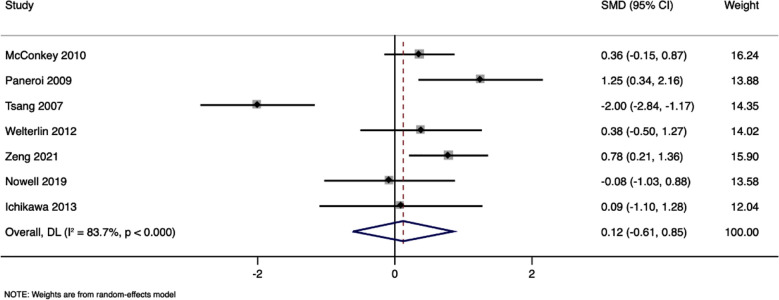
Forest plot showing the difference in socialisation scores between intervention and control arms.

Seven studies with 216 participants have reported socialization scores. Pooled SMD was 0.12 (95%CI, -0.61 to 0.85) with no statistically significant difference between study arms (p=0.74). Fig.3 The I^2^ value was 83.7% indicating a high level of heterogeneity. Sensitivity analysis did not reveal any significant effect on the pooled estimate, indicating robustness of the results.

### Daily living:

**Fig.4:**
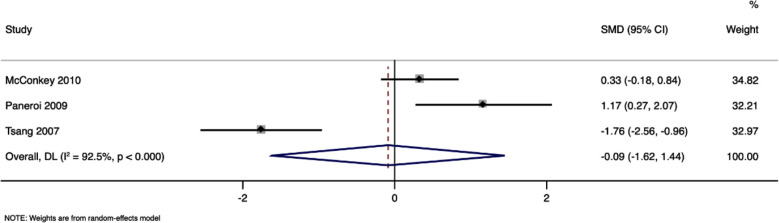
Forest plot showing the difference in daily living scores between intervention and control arms.

Three studies with 118 participants have reported the daily living scores, with pooled SMD of -0.09 (95%CI, -1.62 to 1.44) that was comparable in both study groups (p=0.91) Fig.4 The I^2^ value was 92.5% indicating a high level of heterogeneity. Sensitivity analysis did not reveal any significant effect on the pooled estimate, indicating robustness of the results.

### Motor skills:

**Fig.5:**
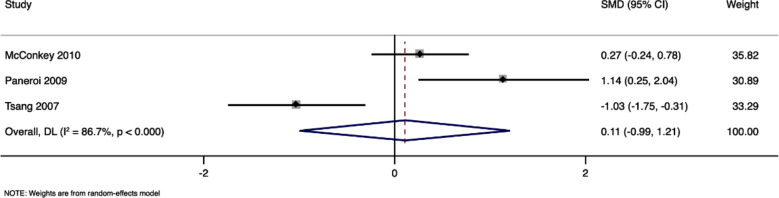
Forest plot showing the difference in motor skills scores between intervention and control arms.

Three studies with 118 participants have reported the motor skills scores in both TEACCH program and standard care groups. Pooled SMD was 0.11 (95%CI, -0.99 to 1.21) and the difference between groups was not statistically significant (p=0.85). Fig.5 The I^2^ value was 86.7% (high heterogeneity). Sensitivity analysis did not reveal any significant effect on the pooled estimate, indicating robustness of the results.

### Adaptive behavior:

Three studies with 134 participants have reported the adaptive behavior scores in both study groups. Pooled SMD was 0.26 (95%CI, -0.59 to 1.12) and it was not statistically significant (p=0.54). The I^2^ value was 81.5% indicating a high level of heterogeneity. Sensitivity analysis did not reveal any significant effect on the pooled estimate, indicating robustness of the results.

### Imitation:

Five studies with 145 participants have reported the imitation scores in both study groups, with pooled SMD of 0.26 (95%CI, -0.28 to 0.80) and no statistical difference between groups (p=0.34). I^2^ value was 59.1%, indicative of the moderate level of heterogeneity.

### Perception:

Four studies with 95 participants have reported perception scores. Pooled SMD was 0.34 (95%CI, -0.07 to 0.75). The scores were comparable in the groups (p=0.11) and no heterogeneity was detected (I^2^ = 0%).

### Fine motor skills:

Five studies encompassing data of 145 participants have reported fine motor skills in children with ASD, participating in TEACCH program and children who received standard care. Pooled SMD was 0.45 (95%CI, 0.12 to 0.79), indicating that the structured teaching program intervention significantly improved fine motor skills of children with ASD when compared to standard care (p=0.007). I^2^ value was 0% indicating no heterogeneity.

### Gross motor skills:

Five studies with 145 participants have reported gross motor skills in both study groups with a pooled SMD of 0.54 (95%CI, 0.21 to 0.88). This shows that the structured teaching program intervention significantly improves gross motor skills when compared to standard care (p=0.001). I^2^ value was 0% indicating no heterogeneity.

### Eye-hand coordination:

Four studies with 95 participants had data of the eye-hand coordination scores in both study groups. Pooled SMD was -0.02 (95%CI, -0.60 to 0.55) with no significant difference between the groups (p=0.94). I^2^ value was 47.7% indicating moderate heterogeneity.

### Cognitive Performance:

Six studies with 164 participants have reported cognitive performance scores in both study groups. Pooled SMD was 0.07 (95%CI, -0.43 to 0.57) and it was not statistically significant (p=0.78). I^2^ value was 57.7% indicating moderate heterogeneity.

### Cognitive verbal performance:

Five studies with 145 participants have reported cognitive verbal performance scores with pooled SMD of 0.03 (95%CI, -0.59 to 0.65) and no intergroup difference (p=0.92). I^2^ value was 68.8% indicating moderate to high level of heterogeneity.

### Parental stress index:

Two studies with 31 participants have assessed parental stress index. Pooled SMD was -0.07 (95%CI, -0.78 to 0.64) and not statistically significant (p=0.85). I^2^ value was 0% indicating no heterogeneity.

## DISCUSSION

This study assessed the efficacy of a structured teaching program, such as TEACCH, for managing ASD in children. The review incorporated a range of outcomes, from communication skills to parental stress and showed that while the structured teaching program shows promise in specific areas, its effects on communication, socialization, daily living skills and cognitive performance were not statistically significant. The only domains with a significant TEACCH program-associated improvement were fine and gross motor skills. This is a novel finding, as most autism interventions do not target motor skills as a primary outcome. This was also in contrast with the previous review findings which showed smaller magnitude of effect sizes.^20^ However, the previous review has incorporated pre-post studies into the meta-analysis which might have introduced certain bias into the findings.^21^ The potential of structured teaching program to specifically enhance motor skills could represent an important consideration for practitioners who aim to provide comprehensive care for children with ASD.^22^

The observed improvements in fine and gross motor skills may stem from the core visual-physical structuring principles of TEACCH. By breaking tasks into discrete, visually cued steps and using clear physical boundaries (e.g., work systems, task boxes, floor mats), children receive constant external cues that reduce cognitive load and support motor planning.^21^,^22^ This hands-on, sequential approach likely enhances procedural learning, allowing children to internalize movement patterns more effectively than in unstructured settings. In addition, the use of visual schedules and tangible prompts may increase engagement and provide immediate feedback, reinforcing motor skill acquisition. Future research could test this hypothesized mechanism by comparing variations in visual support intensity or by incorporating process measures of motor planning and engagement.

Moreover, while the improvements in motor skills are noteworthy, the variable responses in cognitive and adaptive behaviors suggest that structured teaching program may benefit from being paired with other therapeutic modalities. Due to the multidimensional nature of ASD, combining sensory integration therapies or cognitive-behavioral strategies with structured teaching program could potentially amplify its benefits and allow to better address the domains where structured teaching program alone cannot yield significant improvements.^23^

The lack of significant differences between TEACCH program and standard care in several domains raises questions about the differential efficacy of structured teaching program. It is known that ASD presents with a complex array of challenges that may not be uniformly responsive to any one particular intervention. The significant heterogeneity observed in our results suggests that the variability in outcomes may be partially due to individual differences in the ASD population. Moreover, the structured nature of the TEACCH program might be less adaptable to the broad range of learning styles and needs inherent within the autism spectrum, thereby affecting its widespread efficacy.

The methodological quality of the included studies varied, which could have influenced the overall findings. While RCTs generally offer a higher level of evidence, the inclusion of quasi-experimental trials may have introduced bias.^24^ Furthermore, high level of heterogeneity across studies indicates possible variability the way the structured teaching program was implemented, as well as in the populations studied and the outcomes measured. Furthermore, the variations in the delivery of the structured teaching program, such as differences in the intensity, duration and specific components of the intervention, likely contributed to the heterogeneity of the results. The training and expertise of the individuals delivering the program could also have varied, potentially influencing the outcomes. Future research should aim to control for these variables to enhance the comparability of results and provide clearer guidance for practitioners. This heterogeneity limits the generalizability of the results and suggests a need for standardization in future research.

### Limitations:

First, the sample sizes in the included studies were relatively small and the duration of the interventions varied, potentially impacting the efficacy. Second, studies predominantly used outcome measures that may not have been sensitive enough to detect subtle changes, particularly in areas such as socialization and communication, which are complex and multifaceted constructs in ASD. Third, the follow-up periods were often too short to capture long-term outcomes, which are crucial in understanding the enduring impact of structured teaching program. Furthermore, the varying training levels and fidelity of interventionists across studies may have contributed to heterogeneity in treatment delivery and outcome measurement. Additionally, while we included non-randomized (quasi-experimental and observational) designs to capture a broader evidence base, reliance on these study types may limit internal validity and increase susceptibility to confounding bias.

Our analysis indicated no significant heterogeneity for certain outcomes, such as perception and parental stress, suggesting that these areas may be less susceptible to variation across studies and may reflect more consistent domains of improvement. The identification of potential moderators, such as age at intervention onset and baseline severity of ASD symptoms, was not feasible within the scope of this meta-analysis due to insufficient reporting in the primary studies. Future research should aim to clarify these relationships, as they are crucial for tailoring interventions to individual needs.

Although our pooled estimate showed no statistically significant reduction in parental stress, this domain has been assessed in only a handful of trials and remains critically under-explored. Given the substantial caregiver burden associated with ASD, future studies should routinely include validated parental stress measures to determine whether structured teaching programs confer meaningful benefits for family well-being.

Nevertheless, the observed significant improvements in motor skills suggests that structured teaching program may be integrated as a complementary approach in ASD rehabilitation.^25^ It underscores the potential utility of structured teaching program in addressing specific skill deficits within the spectrum of ASD presentations. Our findings also highlight the need to adopt a multifaceted and individualized approach when working with children with ASD. There is a clear need for the development and utilization of sensitive outcome measures that can capture the nuanced changes in ASD symptomatology across multiple domains. Subsequent studies should strive to improve upon the methodological limitations observed in the current body of literature. Larger, more rigorous RCTs with standardized intervention protocols and long-term follow-ups are needed to better evaluate the efficacy of structured teaching program in children with ASD.

### Authors’ contributions:

**YZ:** Study design. Literature search and manuscript writing.

**MZ, DM and PW:** Data collection, data analysis and interpretation. Critical Review.

**YZ:** Manuscript revision and validation and is responsible for the integrity of the study.

All authors have read and approved the final manuscript.

SUPPLEMENTARY APPENDIX

**Search Strategy:**

**Medline (via PubMed):**

(“Autism Spectrum Disorder”[Mesh] OR autism[tiab] OR autistic[tiab] OR ASD[tiab] OR “pervasive developmental disorder”[tiab])

AND

(“structured teaching program”[tiab] OR “structured teaching”[tiab] OR TEACCH[tiab] OR “Treatment and Education of Autistic and Related Communication Handicapped Children”[tiab] OR “visual supports”[tiab] OR “work system”[tiab] OR “individualized schedule”[tiab] OR “environmental structure”[tiab])

AND

(“randomized controlled trial”[pt] OR randomized[tiab] OR randomised[tiab] OR “controlled

clinical trial”[pt] OR “clinical trial”[tiab] OR quasi-experimental[tiab] OR trial[tiab])

**ScienceDirect**:

TITLE-ABSTR-KEY(autism OR “autism spectrum disorder” OR ASD OR “pervasive developmental disorder”)

AND

TITLE-ABSTR-KEY(“structured teaching program” OR TEACCH OR “structured teaching” OR “visual supports” OR “work system” OR “individualized schedule” OR “environmental structure”)

AND

TITLE-ABSTR-KEY(“randomized controlled trial” OR randomized OR randomised OR “controlled trial” OR quasi-experimental OR trial)

**Cochrane CENTRAL**:

([mh “Autism Spectrum Disorder”] OR autism:ti,ab,kw OR ASD:ti,ab,kw OR “pervasive developmental disorder”:ti,ab,kw)

AND

(“structured teaching program”:ti,ab,kw OR TEACCH:ti,ab,kw OR “structured teaching”:ti,ab,kw OR “visual supports”:ti,ab,kw OR “work system”:ti,ab,kw OR “individualized schedule”:ti,ab,kw OR “environmental structure”:ti,ab,kw)

AND

(trial:ti,ab,kw OR randomized:ti,ab,kw OR randomised:ti,ab,kw OR “controlled trial”:ti,ab,kw OR quasi-experimental:ti,ab,kw)

**Google Scholar**:

autism OR “autism spectrum disorder” OR ASD OR “pervasive developmental disorder” “structured teaching program” OR TEACCH OR “structured teaching” OR “visual supports” OR “work system” OR “individualized schedule” OR “environmental structure” “randomized controlled trial” OR randomized OR randomised OR “clinical trial” OR quasiexperimental OR trial.
